# Nutritional Status of Children with Cerebral Palsy—Findings from Prospective Hospital-Based Surveillance in Vietnam Indicate a Need for Action

**DOI:** 10.3390/nu11092132

**Published:** 2019-09-06

**Authors:** Tasneem Karim, Israt Jahan, Rachael Dossetor, Nguyen Thi Huong Giang, Nguyen Thi Van Anh, Trinh Quang Dung, Cao Minh Chau, Nguyen Van Bang, Nadia Badawi, Gulam Khandaker, Elizabeth Elliott

**Affiliations:** 1 Discipline of Child and Adolescent Health, Faculty of Medicine and Health, The University of Sydney, Sydney 2145, Australia; 2 CSF Global, Dhaka 1213, Bangladesh; 3 Asian Institute of Disability and Development (AIDD), University of South Asia, Dhaka 1213, Bangladesh; 4 Cerebral Palsy Alliance Research Institute, The University of Sydney, Sydney 2050, Australia; 5 Rehabilitation Department, National Children’s Hospital, Hanoi 117200, Vietnam; 6 Department of Medical Education and Skills Lab, Hanoi Medical University, Hanoi 116177, Vietnam; 7 Rehabilitation Department, Hanoi Medical University (Bach Mai Hospital), Hanoi 116177, Vietnam; 8 Faculty of Health Sciences, Phenikaa University, Hanoi 152308, Vietnam; 9 Department of Paediatrics, Hanoi Medical University, Hanoi 116177, Vietnam; 10 Faculty of Medicine, Hanoi University of Business and Technology, Hanoi 113400, Vietnam; 11 Central Queensland Public Health Unit, Central Queensland Hospital and Health Service, Rockhampton, Queensland 4700, Australia; 12 The Sydney Children’s Hospitals Network, Westmead, Sydney 2145, Australia

**Keywords:** cerebral palsy, hospital-based surveillance, malnutrition, Vietnam, children

## Abstract

Background: Lack of evidence on the burden and risk factors for malnutrition among children with cerebral palsy (CP) in Vietnam limits evidence-based interventions. We aimed to define the nutritional status of children with CP in Vietnam. Materials and Methods: The study utilized data from active prospective hospital-based surveillance modelled on the Pediatric Active Enhanced Disease Surveillance system. Children (0–18 years) with CP attending the National Children’s Hospital Hanoi, Vietnam between June–November 2017 were included. Data on demographic, clinical and rehabilitation status were collected following detailed neurodevelopmental assessment. Anthropometric measurements were taken. Nutritional status was determined using the World Health Organization guideline. Results: Of 765 children (the mean (SD) age was 2.6 (2.5) years; 35.8% were female), 28.9% (n = 213) were underweight and 29.0% (n = 214) stunted. The odds of underweight were significantly higher among children aged >5 years and/or having a monthly family income of <50 USD. Underweight and/or stunting was high among children with quadriplegia (81%, n = 60 and 84.5%, n = 87) and/or Gross Motor Functional Classification System (GMFCS) level IV–V (62.5%, n = 45 and 67.0%, n = 67). Nearly one-third of intellectually impaired and more than half of hearing-impaired children were underweight and/or stunted. Conclusions: Poor economic status and increased motor severity increased vulnerability to malnutrition. Our findings will inform nutritional rehabilitation programs among these vulnerable children.

## 1. Introduction

Poor nutrition and growth faltering disrupt cognition and development and are commonly observed among children with Cerebral Palsy (CP). CP is a major cause of childhood disability. It is a non-progressive neurological disorder resulting from an insult to the developing brain that affects posture and movement [1]. Oromotor dysfunction and gastrointestinal problems, often associated with motor impairment, hinder optimum nutrition of children with CP [2,3]. The worldwide prevalence of CP is 2.1 per 1000 live births with a rate of 1.4 per 1000 live births observed in Australia [4,5]. However, a recent population-based study in Bangladesh reported a prevalence of 3.4 per 1000 live births [6]. This is substantially higher than the global estimate, which largely reflects findings from high income countries (HICs). This disparity is most likely due to increased risk factors, including birthing practices, in a poor socio-economic context [7,8], which is also a key underlying risk factor for malnutrition among children [9].

Maintenance of optimal nutrition is challenging among children with CP. Although, studies from both HICs and low and middle-income countries (LMICs) have demonstrated high prevalence of malnutrition among children with CP compared to the general population [10,11], evidence suggests that the burden is considerably higher in LMICs than in HICs [10,11,12]. Yet the underlying risk factors in LMICs are poorly understood. Severe motor impairment, measured using the Gross Motor Functional Classification System (GMFCS), presence of associated impairments and oropharyngeal dysphagia affect the nutritional status of children with CP [2,10,12].

Feeding difficulties among children with CP increase their risk of growth failure [3]. A population-based study in Norway reported significantly higher longitudinal growth faltering in the first five years of age among children with CP who had feeding difficulties compared to their peers without such difficulties [11]. In Ghana, children with CP and feeding difficulties had 3 to 10 times higher odds of being underweight than children with CP without feeding difficulties [13]. Similar patterns were observed in Uganda [14]. Moreover, children with CP are at high risk of poor nutritional outcome due to metabolic alterations and changes in body composition for example, decreased fat free mass, changes in body fat level and altered calorie demand, associated with CP [15]. In LMICs, the significance of these risk factors is likely to be intensified due to their complex interaction with socio-economic factors.

Adverse consequences of malnutrition among children with CP are widespread. Evidence from different countries reveals that malnutrition among children with CP results in poor health-related outcomes, poor quality of life and premature mortality [16,17]. The vicious cycle of malnutrition and infection has also been illustrated in several studies [18]. In a recent population-based study in Bangladesh, 86% of deaths among children with CP were attributed to infections and nearly all of the children who died were undernourished [16]. Studies conducted in Bangladesh and Ghana also reported poor quality of life among the caregivers of children with CP and feeding difficulties [13,19]. Existing evidence suggests that the scenario is likely to be similar for children with CP in the resource constraint settings of other LMICs such as Vietnam.

Nutritional interventions such as gastrostomy and tube feeding are beneficial for weight gain among children with CP [20]. However, such interventions are not commonly available or used in low resource settings and are reportedly poorly understood by clinicians and families. Furthermore, in LMICs there is limited access to dieticians and speech pathologists, who play a crucial role in the management of feeding problems among children with CP. Better understanding of the nutritional status of children with CP is essential to guide the development of evidence-based programs suitable for these settings. In this study we aimed to assess the burden of malnutrition and the underlying risk factors among children with CP in Vietnam.

## 2. Materials and Methods

The study utilized data from an active hospital-based surveillance of children with CP in the National Children’s Hospital in Hanoi, Vietnam [21]. The diagnosis of CP was made using the standard definition adopted from Surveillance of Cerebral Palsy in Europe and the Australian CP Register [5,22].

### 2.1. Study Setting and Participants

Study participants included children aged ≤18 years who visited the National Children’s Hospital in Hanoi, Vietnam between June and November 2017 and were diagnosed with CP.

### 2.2. Data Collection and Clinical Assessment

Data were recorded by trained clinicians using a standardized case record form in the Vietnamese language and responses were translated into English by two clinicians on the study team. Sociodemographic factors (e.g., age, gender, income, educational level of the parents), clinical factors (e.g., GMFCS level, Manual Ability Classification System (MACS) level, predominant CP motor type, associated impairments) and anthropometric measurements (e.g., weight, height) were collected as part of the registration and clinical assessment process. A detailed study protocol including the methods and measures used in the study has been published previously [21].

### 2.3. Anthropometric Measurements

We measured height and weight using a standard World Health Organization (WHO) guideline [23].

**Weight:** Weight was measured in kilograms using a digital weighing scale with a precision of 100 g following standard guidelines (i.e., removing extra clothing, standing still). Tared weight was measured for young children aged less than 2 years and for children who could not stand alone. Clinicians were trained before taking anthropometric measurements. Three repeated measures were taken and the average was noted in the questionnaire.

**Height:** Recumbent length was measured in cm for children aged less than 2 years using a length board and standing height was measured for children aged ≥2 years using a standing frame. The mean of three repeated measures were taken to minimize measurement error. For children who could not stand alone, segmental height (i.e., knee height) was measured and full height was derived using the following formula; height = (2.69 × knee height) + 24.2 cm [24].

### 2.4. Data Management and Analysis

Data entry and analyses were carried out using SPSS version 23.0 (IBM Corporation, Chicago, IL, USA). All data were entered into a database by investigators in Sydney and Vietnam. Three indices were used to describe the nutritional status of children with CP—weight for age (WA), height for age (HA) and weight for height (WH). The z scores for these three indices (i.e., WAZ, HAZ and WHZ) were calculated using WHO Anthro (version 3.2) and WHO AnthroPlus software. WAZ was calculated for children aged less than 121 months and WHZ was calculated for children aged less than 61 months. Nutritional status of children was categorized using the WHO cutoffs for the z scores (i.e., −2SD to +2SD: Normal; <−2SD to −3SD: Moderately undernourished and <−3SD: Severely undernourished).

A normality test was conducted for all continuous variables for example, WAZ, HAZ and WHZ using Shapiro Wilk test. In case of variables not normally distributed [WAZ, HAZ, WHZ] we used median [IQR]). Descriptive analyses were done to present socio-demographic characteristics, clinical factors and nutritional status of the study participants. Bivariate analyses were done to identify potential risk factors for malnutrition among children with CP. Chi-square and Fisher’s exact tests were used to examine the statistical differences in nutritional status between groups of children according to their socio-demographic characteristics and clinical factors. The nutritional status of children with CP aged less than five years was compared with the general population of the same age group in Vietnam using World Bank data. Binomial test were done to identify statistically significant differences between the proportion of malnutrition among our study participants and a general population of the same age group. Factors that were found to be related to underweight and/or stunting among children in bivariate analyses for example, chi-square test, Fisher’s exact tests and unadjusted logistic regression were included in an adjusted logistics regression model. A *p* value < 0.05 was considered statistically significant.

### 2.5. Ethical Consideration

Ethics approval was obtained from the University of Sydney Human Research Ethics Committee (HREC) (2016/456), Hanoi Medical University (HMU) (1722/QD-QHYHN) and NCH (812/QD-BVNTU) in Hanoi, Vietnam. Informed consent was obtained from parents or caregivers of all the study participants.

## 3. Results

A total of 765 children with CP and aged less than 18 years was identified between June and November 2017. The mean (SD) age was 2.6 (2.5) years and 35.8% were female.

### 3.1. Overall Nutritional Status

Of the 765 children 71.1% (n = 524) had a normal WAZ, 70.1% (n = 502) had a normal HAZ and 75.1% (n = 435) children had a normal WHZ. The distribution of WAZ, HAZ and WHZ was positively skewed, hence the median [IQR] for these three indicators was reported. The median [IQR] z score for WAZ was −1.3 [−2.2, −0.2], the median z score for HAZ was −1.1 [−2.3, 0.2] and the median z score for WHZ was −0.86 [−2.0, 0.3]. Among the children, 28.9% (n = 213) were underweight, 29.9% (n = 214) were stunted and 24.9% (n = 144) were wasted. When compared to children aged under five years in the general population in Vietnam, children aged less than five years with CP had a significantly higher prevalence of underweight, stunting and wasting (*p* < 0.001 for all three indices). Moreover, 14.4% (n = 103) of children with CP had severe chronic malnutrition (i.e., severe stunting) and 13.8% (n = 80) had severe acute malnutrition (SAM) (i.e., severe wasting). (Table 1)

### 3.2. Socio-Demographic Characteristics and Nutritional Status of Children with CP

Table 2 summarizes the nutritional status of study participants according to their socio-demographic characteristics. A significant inverse relationship was observed between age group and proportion of underweight among children with CP. Among children aged less than two years, 25.3% (*n* = 106) were underweight. The proportion of underweight was significantly higher among children aged 2–4 years (32.3%, *n* = 70) and ≥5 years (36.6%, *n* = 37) (*p* = 0.03). Conversely, stunting was less prevalent among children aged ≥5 years.

The burden of underweight was significantly higher among children who did not have access to safe drinking water compared to those who had access (24.7%, *n* = 101 vs. 41.5%, *n* = 22; *p* = 0.01). Although not statistically significant, a similar finding was observed for stunting among children with CP in our cohort.

Rates of both underweight (39.5%, *n* = 68) and stunting (34.9%, *n* = 59) were highest among children whose monthly family income was less than 50 USD. When compared with the proportion of underweight children in families in our cohort with a monthly family income of >150 USD (21.1%, *n* = 34) the difference was significant (*p* < 0.001). Although stunting was more prevalent among children with a monthly family income of <50 USD compared to those with higher income, this difference was not statistically significant (*p* = 0.23).

### 3.3. Clinical Characteristics and Nutritional Status of Children With CP

#### 3.3.1. Age of CP Diagnosis and Timing of CP

Although not statistically significant, both underweight and stunting were slightly higher among children who had a confirmed diagnosis of CP at or over five years of age than others in the cohort. Similarly, children with pre and perinatally acquired CP had higher rates of underweight and stunting than children with postnatally acquired CP. (Table 3)

#### 3.3.2. Motor Severity and Neurological Type of CP

The proportion of children with underweight was significantly higher among those with GMFCS level V than in children with GMFCS level I (35.8%, *n* = 78 vs. 23.3%, *n* = 38; *p* = 0.04), as was the proportion with stunting (36.8%, *n* = 75 vs. 21.2%, *n* = 36; *p* = 0.01). A similar pattern was observed for MACS level among children aged 4 years and above. Among children with MACS level IV, 41.1% (*n* = 7) were underweight and 35.5% (*n* = 6) were stunted, whereas these percentages were 27.9% (*n* = 17) and 29.4% (*n* = 20) respectively for children with MACS level I (*p* = 0.77 and *p* = 0.58 respectively).

The majority of children in our study had spastic CP, however none had triplegia. Both underweight (31.8%, *n* = 164) and stunting (33.6%, *n* = 166) were more commonly observed among children with quadriplegia than the children without quadriplegia (*p* = 0.01). (Table 3)

#### 3.3.3. Associated Impairments

In our study, 74.4% (*n* = 569) children had one or multiple associated impairments. Among the children who had one or more associated impairments, 33.1% (*n* = 117) were underweight and 34.4% (*n* = 118) were stunted. The proportion with underweight or stunting was significantly higher among children with one or multiple associated impairments (*p* = 0.02 and *p* = 0.01 respectively) than with no associated impairments. Both underweight and stunting were significantly higher among children reported to have intellectual impairment (*p* < 0.001) or hearing impairment (*p* < 0.001) compared to children without these impairments. (Table 3)

#### 3.3.4. Factors Associated with Underweight and Stunting among Children with CP

Table 4 and Table 5 present findings from unadjusted and adjusted analyses. In the adjusted analysis, the odds of being underweight was statistically associated with the age of the children, monthly family income and GMFCS level. Children aged more than five years had 3.2 times higher odds of being underweight than children aged less than two years. When adjusted for other covariates, children from the poorest families (monthly family income <50 USD) had 3.0 times higher odds of being underweight than children whose monthly family income was >150 USD.

Monthly family income was not significantly associated with stunting. However, children between 2 and 4 years of age had higher odds of being stunted than younger children. When adjusted for covariates, the presence of intellectual impairment and motor function at GMFCS levels IV and V significantly increased the odds of stunting. Similarly, underweight was associated with GMFCS levels IV and V but there was no association between intellectual impairment and the level of underweight.

## 4. Discussion

Our novel study findings derive from an active hospital-based surveillance system and provide valuable insights on the burden and risk factors for malnutrition among children with CP in Vietnam. The proportion of children with malnutrition among our study cohort (i.e., 29% underweight and 30% stunted) was significantly higher than that reported in the general child population in Vietnam but lower than in other LMICs [10,14,25]. The reported burden of underweight from recent studies conducted in Bangladesh, Uganda and Ghana was 70%, 42% and 65% respectively [10,13,14] Our study utilized data from a hospital-based surveillance system. Considering the potential risk of representation bias of such a recruitment strategy, our findings may be an underestimate of the true burden of malnutrition among children with CP in Vietnam.

Vietnam has observed a steady improvement in the nutritional status of children over the past decade. The health policy and strategy framework of Vietnam demonstrates a commitment to reducing the burden of malnutrition among young children [26] The National Nutrition Strategy (NNS) of Vietnam (2011–2020) envisions a substantial reduction in malnutrition among children by the year 2020, aligning with the sustainable development goals (SDGs) [27] The cardinal role of nutrition in the maintenance of health and wellbeing is well recognized and is demonstrated further by the emphasis on nutrition in the SDGs, particularly goals 1, 2 and 3. Following the launch of the NNS the Vietnamese government introduced several multisectoral initiatives including nutrition specific and nutrition sensitive intervention programs. The comparatively better nutritional status of children with CP in our study compared to those in other LMICs may be a result of these government initiatives to improve the nutritional outcomes of the population as a whole.

The characteristics of the malnourished children with CP identified through our active surveillance were similar to those from several other LMICs [10,13,14,25]. The majority of children in our study were from younger age groups. The significant association between age and underweight demonstrates the increased vulnerability of these young children to malnutrition as they get older. Similar findings were reported elsewhere [10]. Studies from other countries have also reported the relationship between malnutrition and age and motor severity of CP. Motor severity is likely to be associated with increased severity of oromotor dysfunction among children with CP, which acts as a barrier to self-feeding and/or adequate feeding. Evidence from HICs suggests that children with severe motor impairment for example, GMFCS level IV-V, often face difficulties with oromotor function [28,29]. These children also frequently have feeding difficulties such as choking, drooling, biting and gastro-esophageal reflux [2,3]. All these factors directly hamper regular food intake among these children. Studies also reported that children with severe motor impairment have altered energy requirements and body composition for example, reduced fat free mass, changes in total body fat [30]. This is consistent with our study findings, which revealed a significant association between the nutritional status of children with CP and motor severity and associated impairments.

It is evident that in the absence of appropriate interventions, children with CP are at high risk of developing malnutrition. In HICs, nasogastric tube feeding has been found to be beneficial for weight gain in children with CP [31]. However, this intervention is not widely available or utilized in LMICs, where there is aversion to increased risk of morbidity through adverse consequences for example, overfeeding, infection or aspiration [32]. Studies conducted in Bangladesh and Ghana demonstrate that improved nutritional status among children with CP can be achieved through training of caregivers with a specific focus on recommended feeding behavior [13,19].

Poor feeding practices such as incorrect positioning and nutritional inadequacy of the diet are often closely intertwined with the poor socio-economic status of families of children with CP. In our study, the burden of malnutrition among children with CP is higher among poorer families. This finding remained unchanged when adjustment was made for other socioeconomic and clinical characteristics. A similar pattern was observed in another LMICs [10]. The conceptual framework of malnutrition clearly shows that economic vulnerability directly and indirectly enhances the risk of malnutrition, comorbidity and mortality among children [9].

### Study Limitations

Despite our considerable efforts, this study had limitations. First, it was a hospital-based study which imposed a potential risk for biased representation of children with CP in Vietnam. Thus, the findings must be interpreted with caution and consideration that the true scenario might differ from the reported findings. Second, we focused on selective anthropometric measurements to assess nutritional status. Although, utilization of other inexpensive anthropometric measurements for example, subscapular skinfold thickness, triceps skinfold thickness, mid upper arm circumference could provide more information of the overall nutritional status of children with CP, considering the availability of resources and to minimize errors in the data we only included the height and weight in this study. A detailed assessment, such as measurement of body fat, fat free mass or bone mineral density requires advanced technology for example, biometric impedance analysis, dual-energy X-ray absorptiometry. Assessment of the dietary intake pattern including caregiver’s knowledge, capacity and practice regarding adequate feeding practices to the children with CP and detailed assessment of the level of oromotor dysfunction could potentially yield a better understanding of nutritional status and establish possible causal relationships between risk factors identified in this study for example, socio-economic status, motor severity and malnutrition among children with CP. Third, we assessed the nutritional status of our study participants by comparing their anthropometric data to the WHO reference population. Recent evidence from HICs indicates that the growth curve of children with CP differs from the general population [33]. However, such CP specific and/or ethnicity specific data are not available for LMICs like Vietnam. Nevertheless, the method utilized in our study for the assessment of nutritional status is widely accepted in these settings.

## 5. Conclusions

Global evidence shows that malnutrition increases the risk of morbidity and mortality among children. This is intensified when malnutrition is accompanied by a disability such as CP. Although evidence from different countries has identified few common risk factors for malnutrition among children with CP, the burden and intensity is highly influenced by contextual factors. Considering the dearth of available evidence from LMICs, our study has generated new data that add substantially to current knowledge about the nutritional status of children with CP in Vietnam. These findings will guide the development of evidence-based training for health professionals and interventions for children with CP in Vietnam.

## Figures and Tables

**Table 1:** Nutritional status of the study participants.

Indicators	Children with CP in Our Study			General Population (Aged <5 Years) in 2015 ^1^	*p* Value ^2^
	Overall		Aged <5 Years		
	Median [IQR]	n (%)	n (%)	(%)	
**Weight-for-age z score (*n* = 737) ^3^**					
Normal	−1.25 [−2.2, −0.2]	524 (71.1)	−	−	**<0.001**
Underweight					
Moderate		139 (18.9)	176 (27.7)	14.1	
Severe		74 (10.0)			
**Height-for-age z score (*n* = 716) ^4^**					
Normal	−1.1 [−2.3, 0.2]	502 (70.1)	−	−	**<0.001**
Stunted					
Moderate		111 (15.5)	184 (30.6)	24.6	
Severe		103 (14.4)			
**Weight-for-height z score (*n* = 579) ^5^**					
Normal	−0.86 [−2.0, 0.3]	435 (75.1)	−	−	**<0.001**
Wasted					
Moderate		64 (11.1)	142 (24.7)	6.4	
Severe		80 (13.8)			

^1^ The World Bank data, 2015. Available from: https://data.worldbank.org. ^2^ Binomial test. ^3^ Weight-for-age z score was calculated for children aged ≤121 months. ^4^ Missing data (n = 49). ^5^ Weight-for-height z score was calculated for children aged ≤61 months. The *p* values for statistically significant differences are shown in bold.

**Table 2:** Socio-demographic characteristics of children and their nutritional status.

Indicators	Total	Weight-for-Age Z Score (*n* = 737) ^1^			Height-for-Age Z Score (*n* = 716) ^2^		
	*n* = 765	Normal *n* = 524	Underweight *n* = 213	*p* Value ^3^	Normal *n* = 502	Stunted *n* = 214	*p* Value ^3^
**Age groups (years), *n* = 765**							
less than 2	424 (55.4)	313 (74.7)	106 (25.3)	**0.03**	282 (72.3)	108 (27.7)	0.07
2–4	220 (28.8)	147 (67.7)	70 (32.3)		135 (64.0)	76 (36.0)	
5 and more	121 (15.8)	64 (63.4)	37 (36.6)		85 (73.9)	30 (26.1)	
**Sex, *n* = 765**							
Female	274 (35.8)	200 (75.2)	66 (24.8)	0.07	184 (71.6)	73 (24.8)	0.52
Male	491 (64.2)	324 (68.8)	147 (31.2)		318 (69.3)	141 (30.7)	
**Source of drinking water, *n* = 761**							
Piped water	423 (55.6)	308 (75.3)	101 (24.7)	**0.01**	287 (72.8)	107 (27.2)	0.17
Well water	284 (37.3)	182 (66.9)	90 (33.1)		179 (66.8)	89 (33.2)	
Other sources (ponds/river/stream/lake)	54 (7.1)	31 (58.5)	22 (41.5)		33 (64.7)	18 (35.3)	
**Sanitation practice, *n* = 759**							
Flush toilet	633 (83.4)	439 (72.1)	170 (27.9)	0.18	425 (71.7)	168 (28.3)	0.04
Pit toilet	126 (16.6)	82 (66.1)	42 (33.9)		74 (62.2)	45 (37.8)	
**Monthly family income, *n* = 728**							
below 50 USD	180 (24.7)	104 (60.5)	68 (39.5)	**0.002**	110 (65.1)	59 (34.9)	0.23
51-100 USD	231 (31.7)	158 (71.8)	62 (28.2)		154 (70.0)	66 (30.0)	
101-150 USD	153 (21.0)	109 (73.6)	39 (26.4)		103 (71.5)	41 (28.5)	
Above 150 USD	164 (22.5)	127 (78.9)	34 (21.1)		112 (75.7)	36 (24.3)	

^1^ Weight-for-age z score was calculated for children aged ≤121 months. ^2^ Missing data (n = 49). ^3^ Chi square test. The *p* values for statistically significant differences are shown in bold.

**Table 3:** Clinical characteristics and nutritional status of the children with Cerebral Palsy (CP).

Indicators	Total	Weight-for-Age Z Score, (*n* = 737) ^1^			Height-for-Age Z Score, (*n* = 716) ^2^		
	*n* = 765	Normal *n* = 524 n (%)	Underweight *n* = 213 n (%)	*p* Value ^3^	Normal *n* = 502 n (%)	Stunted *n* = 214 n (%)	*p* Value ^3^
**Age of CP diagnosis (years), *n* = 754**							
less than 2	559 (74.1)	395 (72.2)	152 (27.8)	0.45	370 (70.7)	153 (29.3)	0.87
2–4	143 (19.0)	95 (68.3)	44 (31.7)		94 (68.6)	43 (31.4)	
5 and more	52 (6.9)	28 (65.1)	15 (34.9)		33 (68.8)	15 (31.3)	
**Timing of CP, *n* = 753**							
Postnatal	98 (13.0)	72 (75.0)	24 (25.0)	0.44	72 (76.6)	22 (23.4)	0.05
Pre and perinatal	416 (55.2)	279 (69.4)	123 (30.6)		259 (66.4)	131 (33.6)	
Unknown	239 (31.7)	167 (72.9)	62 (27.1)		165 (74.0)	58 (26.0)	
**GMFCS level, *n* = 754**							
I	179 (23.7)	125 (76.7)	38 (23.3)	**0.04**	134 (78.8)	36 (21.2)	**0.01**
II	121 (16.0)	89 (76.7)	27 (23.3)		80 (69.6)	35 (30.4)	
III	103 (13.7)	76 (73.8)	27 (26.2)		71 (75.5)	23 (24.5)	
IV	131 (17.4)	88 (68.8)	40 (31.3)		81 (65.9)	42 (34.1)	
V	220 (29.2)	140 (64.2)	78 (35.8)		129 (63.2)	75 (36.8)	
**MACS level, *n* = 188**							
I	71 (37.8)	44 (72.1)	17 (27.9)	0.77	48 (70.6)	20 (29.4)	0.58 ^4^
II	73 (38.8)	47 (69.1)	21 (30.9)		48 (69.6)	21 (30.4)	
III	26 (13.8)	17 (70.8)	7 (29.2)		18 (75.0)	6 (25.0)	
IV	17 (9.0)	10 (58.8)	7 (41.1)		11 (64.7)	6 (35.3)	
V	1 (0.5)	-	-		0 (0.0)	1 (100.0)	
**Neurological type of CP, *n* = 765**							
Mono/hemi	157 (20.5)	118 (79.2)	31 (20.8)	0.10^4^	119 (79.9)	30 (20.1)	**0.01 ^4^**
Diplegia	40 (5.2)	30 (76.9)	9 (23.1)		29 (76.3)	9 (23.7)	
Triplegia	0 (0.0)	0 (0.0)	0 (0.0)		0 (0.0)	0 (0.0)	
Quadriplegia	532 (69.5)	352 (68.2)	164 (31.8)		328 (66.4)	166 (33.6)	
Ataxia	1 (0.1)	1 (100.0)	0 (0.0)		0 (0.0)	1 (100.0)	
Dyskinesia	35 (4.6)	23 (71.9)	9 (28.1)		26 (76.5)	8 (23.5)	
**Number of associated impairments, *n* = 765**							
None	196 (25.6)	143 (78.1)	40 (21.9)	**0.02**	146 (78.5)	40 (21.5)	**0.01**
At least one	206 (26.9)	145 (72.1)	56 (27.9)		131 (70.1)	56 (29.9)	
Multiple	363 (47.5)	236 (66.9)	117 (33.1)		225 (65.6)	118 (34.4)	
**Type of associated impairments**							
Epilepsy	82 (10.8)	54 (68.4)	25 (31.6)	0.41	56 (71.8)	22 (28.2)	0.71
Intellectual	439 (65.6)	283 (66.6)	142 (33.4)	**0.001**	273 (65.5)	144 (34.5)	**<0.001**
Visual	40 (5.4)	24 (61.5)	15 (38.5)	0.11	21 (53.8)	18 (46.2)	**0.02**
Hearing	13 (1.7)	6 (46.2)	7 (53.8)	**0.04**	9 (69.2)	4 (30.8)	0.60 ^4^
Speech	449 (63.7)	305 (69.2)	136 (30.8)	0.07	281 (67.9)	133 (32.1)	**0.04**
**Swallowing difficulties, *n* = 740**							
Present	60 (8.1)	39 (7.7)	20 (9.6)	0.39	38 (7.8)	18 (8.8)	0.64
Absent	680 (91.9)	469 (92.3)	188 (90.4)		451 (92.2)	186 (91.2)	
**Received rehabilitation services, *n* = 759**							
*Ever received rehabilitation services*	703 (92.6)	483 (70.9)	198 (29.1)	0.56	461 (69.1)	206 (30.9)	**0.03**

^1^ Weight-for-age z score was calculated for children aged ≤121 months. ^2^ Missing data (***n*** = 49). ^3^ Chi square test. ^4^ Fisher’s exact test. The *p* values for statistically significant differences are shown in bold.

**Table 4:** Factors associated with underweight and stunting among children with CP in Vietnam (unadjusted analyses).

Covariates	Underweight		Stunting	
	Odds Ratio (95% CI) (Unadjusted)	*p* Value	Odds Ratio (95% CI) (Unadjusted)	*p* Value
**Age group (years)**				
less than 2	*Reference*			
2–4	1.4 (1.0, 2.0)	0.63	1.5 (1.2, 2.1)	**0.03**
5 and more	1.7 (1.1, 2.7)	**0.02**	0.9 (0.6, 1.5)	0.73
**Source of drinking water**				
Well water	*Reference*			
Piped water	0.7 (0.5, 0.9)	**0.02**	0.7 (0.5, 1.0)	0.09
Other sources (ponds/river/stream/lake)	1.4 (0.8, 2.6)	0.24	1.1 (0.6, 2.5)	0.77
**Monthly family income (USD)**				
Above 150	*Reference*			
below 50	2.4 (1.5, 4.0)	**<0.001**	1.7 (1.0, 2.7)	0.04
51–100	1.5 (0.9, 2.4)	0.12	1.3 (0.8, 2.1)	0.23
101–150	1.3 (0.8, 2.3)	0.28	1.2 (0.7, 2.1)	0.42
**GMFCS level**				
I	*Reference*			
II	1.0 (0.6, 1.7)	0.99	1.6 (0.9, 2.8)	0.08
III	1.2 (0.7, 2.1)	0.59	1.2 (0.7, 2.2)	0.54
IV	1.5 (0.9, 2.5)	0.13	1.9 (1.1, 3.2)	**0.01**
V	1.8 (1.2, 2.9)	**0.01**	2.2 (1.3, 3.4)	**0.001**
**Intellectual impairment**				
No	*Reference*			
Yes	0.5 (0.4, 0.8)	**0.001**	0.5 (0.3, 0.7)	**<0.001**
**Hearing impairment**				
No	*Reference*			
Yes	0.3 (0.1, 1.0)	0.05	0.9 (0.3, 3.1)	0.93
**Visual impairment**				
No	*Reference*			
Yes	0.6 (0.3, 1.2)	0.17	0.5 (0.2, 0.9)	**0.02**
**Speech Impairment**				
No	*Reference*			
Yes	0.7 (0.5, 1.1)	0.12	0.7 (0.5, 1.0)	0.04
**Neurological type of CP ^1^**				
Mono/hemiplegia	*Reference*			
Diplegia	1.1 (0.5, 2.6)	0.76	1.2 (0.5, 2.9)	0.63
Quadriplegia	1.8 (1.1, 2.7)	0.01	2.0 (1.3, 3.1)	0.002
Dyskinesia	1.5 (0.6, 3.5)	0.37	1.2 (0.5, 3.0)	0.66

^1^ There were no triplegic child and one Ataxic child in our cohort. Due to the smaller size, we have excluded these two categories from this unadjusted analysis. The *p* values for statistically significant differences are shown in bold.

**Table 5:** Factors associated with underweight and stunting among children with CP in Vietnam (unadjusted analysis).

Covariates	Underweight		Stunting	
	Odds Ratio (95% CI) (Adjusted)	*p* Value	Odds Ratio (95% CI) (Adjusted)	*p* Value
**Age group (years)**				
less than 2	*Reference*			
2–4	2.1 (1.3, 3.5)	**0.002**	2.0 (1.3, 3.2)	**0.003**
5 and more	3.1 (1.7, 6.1)	**0.001**	1.2 (0.6, 2.4)	0.58
**Source of drinking water**				
Well water	*Reference*			
Piped water	0.9 (0.5, 1.3)	0.52	0.8 (0.5, 1.3)	0.47
Other sources (ponds/river/stream/lake)	1.4 (0.7, 2.9)	0.38	1.2 (0.5, 2.5)	0.65
**Monthly family income (USD)**				
Above 150	*Reference*			
below 50	3.0 (1.5, 5.7)	**0.001**	1.3 (0.7, 2.4)	0.43
51–100	1.8 (1.0, 3.4)	0.06	1.2 (0.7, 2.2)	0.50
101–150	1.4 (0.7, 2.8)	0.34	1.1 (0.5, 2.0)	0.85
**GMFCS level**				
I	*Reference*			
II	1.3 (0.7, 2.6)	0.40	1.7 (0.9, 3.2)	0.11
III	1.6 (0.7, 3.4)	0.23	1.0 (0.4, 2.1)	0.93
IV	2.9 (1.4, 6.0)	**0.005**	1.9 (0.9, 4.0)	**0.08**
V	2.4 (1.1, 4.9)	**0.02**	2.1 (1.0, 4.3)	**0.04**
**Intellectual impairment**				
No	*Reference*			
Yes	1.3 (0.7, 2.3)	0.35	2.0 (1.1, 3.5)	**0.02**
**Hearing impairment**				
No	*Reference*			
Yes	1.9 (0.5, 7.3)	0.35	1.3 (0.3, 4.9)	0.72
**Visual impairment**				
No	*Reference*			
Yes	1.5 (0.6, 3.4)	0.35	1.6 (0.7, 3.6)	0.26
**Speech Impairment**				
No	*Reference*			
Yes	0.8 (0.5, 1.4)	0.48	0.7 (0.4, 1.1)	0.13
**Neurological type of CP ^1^**				
Mono/hemiplegia	*Reference*			
Diplegia	1.2 (0.4, 3.2)	0.70	1.2 (0.4, 3.3)	0.69
Quadriplegia	1.2 (0.7, 2.1)	0.01	1.4 (0.8, 2.5)	0.23
Dyskinesia	1.3 (0.4, 3.9)	0.68	1.8 (0.6, 5.4)	0.30

^1^ There were no triplegic child and one Ataxic child in our cohort. Due to the smaller size, we have excluded these two categories from this unadjusted analysis. The *p* values for statistically significant differences are shown in bold.

## References

[1] Rosenbaum P., Paneth N., Leviton A., Goldstein M., Bax M., Damiano D., Dan B., Jacobsson B. (2007). A report: The definition and classification of cerebral palsy April 2006. Dev. Med. Child Neurol. Suppl..

[2] Aggarwal S., Chadha R., Pathak R. (2015). Nutritional status and growth in children with cerebral palsy: A review. Int. J. Med. Sci. Public Health.

[3] Aggarwal S., Chadha R., Pathak R. (2015). Feeding Difficulties among Children with Cerebral Palsy: A Review. Int. J. Health Sci. Res..

[4] Oskoui M., Coutinho F., Dykeman J., Jetté N., Pringsheim T. (2013). An update on the prevalence of cerebral palsy: A systematic review and meta-analysis. Dev. Med. Child Neurol..

[5] ACPR Report of the Australian Cerebral Palsy Register, Birth Years 1995–2012. Australia. https://www.ausacpdm.org.au/resources/australian-cerebral-palsy-register/.

[6] Khandaker G., Muhit M., Karim T., Smithers-Sheedy H., Novak I., Jones C., Badawi N. (2019). Epidemiology of Cerebral Palsy (CP) in Bangladesh: Findings from the first population-based CP register for children in a low-and middle-income country. Dev. Med. Child Neurol..

[7] Forthun I., Strandberg-Larsen K., Wilcox A.J., Moster D., Petersen T.G., Vik T., Lie R.T., Uldall P., Tollånes M.C. (2018). Parental socioeconomic status and risk of cerebral palsy in the child: Evidence from two Nordic population-based cohorts. Int. J. Epidemiol..

[8] Durkin M.S. (2016). Socio-economic disparities and functional limitations of children with cerebral palsy. Dev. Med. Child Neurol..

[9] UNICEF (1998). The State of World’s Children. https://www.unicef.org/sowc/archive/ENGLISH/The%20State%20of%20the%20World%27s%20Children%201998.pdf.

[10] Jahan I., Muhit M., Karim T., Smithers-Sheedy H., Novak I., Jones C., Badawi N., Khandaker G. (2019). What makes children with cerebral palsy vulnerable to malnutrition? Findings from the Bangladesh cerebral palsy register (BCPR). Disabil. Rehabil..

[11] Strand K.M., Dahlseng M.O., Lydersen S., Ro T.B., Finbraten A.K., Jahnsen R.B., Andersen G.L., Vik T. (2016). Growth during infancy and early childhood in children with cerebral palsy: A population-based study. Dev. Med. Child Neurol..

[12] Perenc L., Przysada G., Trzeciak J. (2015). Cerebral palsy in children as a risk factor for malnutrition. Ann. Nutr. Metab..

[13] Polack S., Adams M., O’banion D., Baltussen M., Asante S., Kerac M., Gladstone M., Zuurmond M. (2018). Children with cerebral palsy in Ghana: Malnutrition, feeding challenges and caregiver quality of life. Dev. Med. Child Neurol..

[14] Kakooza-Mwesige A., Tumwine J.K., Eliasson A.C., Namusoke H.K., Forssberg H. (2015). Malnutrition is common in Ugandan children with cerebral palsy, particularly those over the age of five and those who had neonatal complications. Acta Paediatr..

[15] Sung K.H., Chung C.Y., Lee K.M., Cho B.C., Moon S.J., Kim J., Park M.S. (2017). Differences in Body Composition According to Gross Motor Function in Children with Cerebral Palsy. Arch. Phys. Med. Rehabil..

[16] Power R., King C., Muhit M., Heanoy E., Galea C., Jones C., Badawi N., Khandaker G. (2018). Health-related quality of life of children and adolescents with cerebral palsy in low-and middle-income countries: A systematic review. Dev. Med. Child Neurol..

[17] Jahan I., Karim T., Das M.C., Muhit M., Mcintyre S., Smithers-Sheedy H., Badawi N., Khandaker G. (2019). Mortality in children with cerebral palsy in rural Bangladesh: A population-based surveillance study. Dev. Med. Child Neurol..

[18] Karim T., Muhit M., Khandaker G. (2017). Interventions to prevent respiratory diseases-Nutrition and the developing world. Paediatr. Respir. Rev..

[19] Adams M.S., Khan N.Z., Begum S.A., Wirz S.L., Hesketh T., Pring T.R. (2012). Feeding difficulties in children with cerebral palsy: Low-cost caregiver training in Dhaka, Bangladesh. Child Care Health Dev..

[20] Ferluga E.D., Sathe N.A., Krishnaswami S., Mcpheeters M.L. (2014). Surgical intervention for feeding and nutrition difficulties in cerebral palsy: A systematic review. Dev. Med. Child Neurol..

[21] Khandaker G., Van Bang N., Dũng T.Q., Giang N.T., Chau C.M., Van Anh N.T., Van Thuong N., Badawi N., Elliott E.J. (2017). Protocol for hospital based-surveillance of cerebral palsy (CP) in Hanoi using the Paediatric Active Enhanced Disease Surveillance mechanism (PAEDS-Vietnam): A study towards developing hospital-based disease surveillance in Vietnam. BMJ Open.

[22] Cans C. (2000). Surveillance of cerebral palsy in Europe: A collaboration of cerebral palsy surveys and registers. Dev. Med. Child Neurol..

[23] World Health Organization (1997). WHO Global Database on Child Growth and Malnutrition.

[24] Stevenson R.D. (1995). Use of segmental measures to estimate stature in children with cerebral palsy. Arch. Pediatr. Adolesc. Med..

[25] Caram A.L.A., Morcillo A.M., Costa-Pinto E.A.L. (2008). Nutritional status of children with cerebral palsy in a Brazilian tertiary-care teaching hospital. Dev. Med. Child Neurol..

[26] (2017). World Bank Data. https://data.worldbank.org/indicator/SH.STA.STNT.ZS?view=chart.

[27] The National Nutrition Strategy for 2011–2020, with a Vision Toward 2030. https://extranet.who.int/nutrition/gina/sites/default/files/VNM%202011%202.%20National%20Nutrition%20%20Strategy%202011-2020.pdf.

[28] Herrera-Anaya E., Angarita-Fonseca A., Herrera-Galindo V.M., Martinez-Marin R.D.P., Rodriguez-Bayona C.N. (2016). Association between gross motor function and nutritional status in children with cerebral palsy: A cross-sectional study from Colombia. Dev. Med. Child Neurol..

[29] Dahlseng M.O., Finbråten A.K., Júlíusson P.B., Skranes J., Andersen G., Vik T. (2012). Feeding problems, growth and nutritional status in children with cerebral palsy. Acta Paediatr..

[30] Benfer K.A., Weir K.A., Bell K.L., Ware R.S., Davies P.S., Boyd R.N. (2015). Food and fluid texture consumption in a population-based cohort of preschool children with cerebral palsy: Relationship to dietary intake. Dev. Med. Child Neurol..

[31] Brooks J., Day S., Shavelle R., Strauss D. (2011). Low weight, morbidity and mortality in children with cerebral palsy: New clinical growth charts. Pediatr. Eng. Ed..

[32] Bell K.L., Samson-Fang L. (2013). Nutritional management of children with cerebral palsy. Eur. J. Clin. Nutr..

[33] Henderson R.C., Grossberg R.I., Matuszewski J., Menon N., Johnson J., Kecskemethy H.H., Vogel L., Ravas R., Wyatt M., Bachrach S.J. (2007). Growth and nutritional status in residential center versus home-living children and adolescents with quadriplegic cerebral palsy. J. Pediatr..

